# Differential Metabolomics and Network Pharmacology Analysis of Silkworm Biotransformation between Mulberry Leaves and Silkworm Droppings

**DOI:** 10.1155/2021/8819538

**Published:** 2021-06-29

**Authors:** Mingqian Li, Lin Chen, Yuntao Dai, Jiacheng Li, Fei Li, Qun Li, Zhihong Yu, Kequn Chai, Yongqiang Zhu

**Affiliations:** ^1^Cancer Institute of Integrated Tradition Chinese and Western Medicine, Zhejiang Academy of Traditional Chinese Medicine, Tongde Hospital of Zhejiang Province, Hangzhou, Zhejiang 310012, China; ^2^Sericultural Research Institute, Zhejiang Academy of Agricultural Sciences, Hangzhou 310021, China; ^3^Institute of Chinese Materia Medica, China Academy of Chinese Medical Sciences, Beijing 100700, China; ^4^College of Life Science, Sichuan Normal University, Chengdu, Sichuan 610101, China

## Abstract

Silkworm droppings are the product of mulberry leaves digested by silkworm intestines, which are an important medicinal resource in traditional Chinese medicine (TCM). The contents of total fat, fat acids, crude protein, amino acids, and secondary metabolites of obtained mulberry leaves and silkworm droppings were analyzed by HPLC, GC-MS, and UHPLC-Q-TOF MS. The target genes and enriched pathways related to significantly changed compositions between mulberry leaves and silkworm droppings were analyzed by network pharmacology. High unsaturated C18 : 3 fatty acids were transformed to low unsaturated C18 : 1 from mulberry leaves to silkworm droppings. Only lysine and 17 mini-peptides had significantly higher content in silkworm droppings than in mulberry leaves. There were 36 common target genes or the different compounds between mulberry leaves and silkworm droppings. The main pathways of mulberry leaf were enriched in antivirus and anticancer properties, while the pathways of silkworm droppings were enriched in hormone regulation and signal transduction.

## 1. Introduction

Traditional Chinese medicine has an extensive history and has been applied to prevent and cure diverse diseases in China and Asian countries [1]. Mulberry leaves (ML, *Morus alba* L.) and silkworm droppings (SD) are important medicinal resources of traditional Chinese medicine. ML play a pivotal role in the sericulture industry because they are the sole food of silkworm (*Bombyx mori* L.) [2]. When the silkworm ingests ML, about 60% of the leaves were excreted without digestion, resulting in droppings (also known as Can-sha in China) that are composed of both mulberry leaf material and various materials transformed by enzymes and microbes in the intestine of the silkworm [3]. In traditional Chinese medicine, ML is used for dispelling wind-heat, moistening the lungs, soothing the liver, and brightening the eyes, while silkworm droppings are used to expel wind, harmonize the stomach, transform turbidity, and disperse dampness, as well as activating blood and promoting menstruation. The different efficacy of them may be caused by the metabolism of silkworms. The cold/hot natures of them were changed from cool to warm. The cold/hot natures theory of Chinese materia medica is one of the essential and foundational principles in traditional Chinese medicine (TCM) and clinical therapy [4]. Biotransformation plays an important role in this process, such as fermentation and biotransformation of tissues and organs [5].

*Morus alba* L., widely distributed in tropical, subtropical, and temperate areas, is an excellent source of nutrients and phytochemicals [6]. ML are a precious source of macro- and micronutrients and organic acids [7]. ML contain fatty acids, amino acids, polysaccharides, flavonoids, alkaloids, volatile oils, and other active compounds with good antioxidation, antibacterial, anti-inflammatory, hypoglycemic, and lipid-lowering roles [8–12]. The ML tea is rich in *γ*-aminobutyric acid (2.7 mg g^−1^ dry weight) which is 10 times higher than that of green tea [13]. The chemical constituents of silkworm dropping are major in chlorophyll and chlorophyll derivatives, xanthophyll, carotenoid, flavonoids, and so forth [14, 15]. Besides, the compositions of the lipids in SD were concentrated liposoluble compounds such as phytosterol, unsaturated fatty acid, and fatty alcohol [16]. The ML and SD are multicomponent, multitarget, and multipathway [17]. However, current understanding of different pharmacology and mechanism of them is limited.

Recently, more and more approaches have been studied to identify the characteristics of Chinese medicinal materials, including chemical metabolites, network pharmacology, and bioinformation [18–21]. The HPLC, GC-MS, and UHPLC-Q-TOF MS are important techniques for the quality evaluation of natural products [22–24]. In our research, the different pharmacology and composition of ML and SD were analyzed by differential metabolomics (HPLC, GC-MS, and UHPLC-Q-TOF MS) and network pharmacology. The contents of various long-chain fatty acids and amino acids were significantly changed. 386 compounds were found to differ in ML and SD. The target genes of high content compounds in ML were mainly enriched in virus infection and cancer signaling pathways. The target genes of high content compounds in SD were mainly enriched in neuroactive ligand-receptor interaction, bile secretion and insulin resistance, and signaling pathway. The results can provide new insight into the pharmacology and pharmacodynamics of ML and SD.

## 2. Materials and Methods

### 2.1. Sample Preparation for Metabolomics Study

Mulberry leaves (*Morus alba*) were harvested from a mulberry garden of Zhejiang University in Hangzhou, Zhejiang Province, China. Each leaf was symmetrically divided into two parts. Half of the leaves were dried at 40°C and ground to a fine powder in an electric grinder. The other half of leaves were fed to the fifth instar third-day silkworm (Qiufeng × Baiyu) to get the intraday SD. The obtained silkworm droppings were dried at 40°C and ground to a fine powder in an electric grinder. The powders of mulberry leaves and silkworm droppings were stored in a biobank of Zhejiang Academy of Traditional Chinese Medicine.

### 2.2. Crude Fat and Fatty Acids Content Determination

The 1.0 g sample was wrapped in a filter paper tube and then put into the Soxhlet extractor. Petroleum ether was added and extracted in a water bath at 40°C for 6–8 h. After the extraction, recycling extract and filter paper tubes were dried and weighed to get total fat content. The 1 g sample was soaked overnight in 2 mL of petroleum ether, 2 mL of n-hexane, and 2 mL of 0.4 m KOH/CH_3_OH solution. The saturated salt solution was added and stratified, and the upper extract was dried and redissolved by n-hexane. The 1 *µ*L fatty acids extract was analyzed by GC-MS.

### 2.3. Crude Protein and Amino Acids Content Determination

The 0.5 g sample, 0.4 g CuSO_4_, 6 g K_2_SO_4_, and 20 mL H_2_SO_4_ were added to the digester and digested at 420°C for 1 h. After cooling, 50 mL water was added to the sample to conduct titration by semiautomatic Kjeldahl nitrogen meter to calculate the crude protein content. The 0.5 g sample was hydrolyzed at 110°C for 22 h with 10 ml 6 N HCl. The upper extract was dried and redissolved by 2 mL 0.1 N HCl. The content of amino acid was determined by liquid chromatography.

### 2.4. UHPLC-MS Analysis

A 100 mg aliquot of the sample was extracted with 1000 *μ*L methanol/water mixture (*v* : *v* = 3 : 1) overnight at 4°C on a shaker and centrifuged at 12500 rpm for 15 min at 4°C. The UHPLC separation was carried out using a Waters ACQUITY UPLC HSS T3 column (100 × 2.1 mm, 1.8 *μ*m). Mobile phase A was 0.1% formic acid in the water, and mobile phase B was acetonitrile. The column temperature was set at 40°C. The autosampler temperature was set at 4°C and the injection volume was 2 *μ*L.

The QE Focus mass spectrometer was used for its ability to acquire MS/MS spectra on an information-dependent basis (IDA) during an LC/MS experiment. In this mode, the acquisition software (Xcalibur 4.1, Thermo) continuously evaluates the full-scan survey MS data as it collects and triggers the acquisition of MS/MS spectra depending on preselected criteria. In each cycle, 3 precursor ions whose intensity was greater than 5000 were chosen for fragmentation at collision energy. Acquired mass range was divided into 70–300, 290–600, and 590–1100 with 3 injections. ESI source conditions were set as follows: spray voltage: +3500/-3500 V, capillary temperature: 350 °C, sheath gas:30, aux gas:10, CE: 10, 30, and 50. AB Sciex QTrap 6500 mass spectrometer was applied for assay development. Typical ion source parameters were ion spray voltage: +5500/−4500 V, curtain gas: 35 psi, temperature: 550°C, ion source gas 1 : 60 psi, ion source gas 2 : 55 psi, and DP: ±100V.

### 2.5. UHPLC-MS Data Preprocessing, Annotation, and Different Compounds Analysis

The high-resolution MS data were converted to the mzXML format using ProteoWizard and processed by MAPS software (version 1.0). The preprocessing results generated a data matrix that consisted of the retention time (RT), mass-to-charge ratio (m/z) values, and peak intensity. In-house MS2 database was applied in metabolites identification; and the MRM data were processed with Skyline software. After being recognized and aligned, the resultant datasets were analyzed to conduct multivariate statistical analysis by SIMCA-P software package, including supervised partial least squares discrimination analysis (PLS-DA).

Parameters including *R2* and *Q2* (cum) were used to assess the quality of PLS-DA and orthogonal partial least squares discriminant analysis (OPLS-DA) models [25]. The differentiating features were extracted by the variable importance in the projection (VIP) values (VIP > 1.0, *P* < 0.05).

### 2.6. Network Pharmacology Analysis

To determine the bioactivity of different chemical components between mu ML and SD, we performed a database search by SymMap (https://www.symmap.org/) and Traditional Chinese Medicine Systems Pharmacology (TCMSP) (http://tcmspw.com/tcmsp.php) using different chemical components. The target genes of different chemical components were found. The Gene Ontology (GO) function and Kyoto Encyclopedia of Genes and Genomes (KEGG) pathway of target genes were enriched by clusterProfiler. The component-target-pathway interaction was established through Cytoscape.

## 3. Results

### 3.1. Different Content Analysis of Crude Fats and Fatty Acids

**Figure 1:**
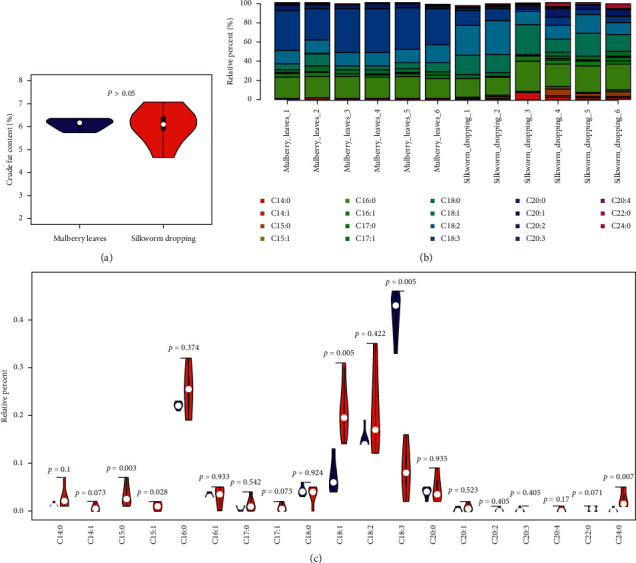
Total fat and fatty acid content in ML and SD. (a) Total fat content in ML and SD. (b) Bar plot of 19 fatty acids proportions in ML and SD. (c) Violin plot of 19 fatty acids proportions in ML and SD. The samples of ML were blue and samples of SD were red.

Fats play a significant role in human health and nutrition. Fats store energy in the body and are transported to fat-soluble vitamins in the blood. We found that the crude fats contents of ML and SD had no significant differences (*P* > 0.05) (Figure 1(a) and Table S1). However, the contents of long-chain fatty acids including C15 : 0, C15 : 1, C18 : 1, and C24 : 0 in SD were significantly higher than those in ML (*P* < 0.05) (Figure 1 and Table S1). The content of C18 : 3 fatty acids in SD was significantly lower than that in ML (*P* < 0.05) (Figure 1 and Table S1).

### 3.2. Different Content Analysis of Crude Protein and Amino Acids

**Figure 2:**
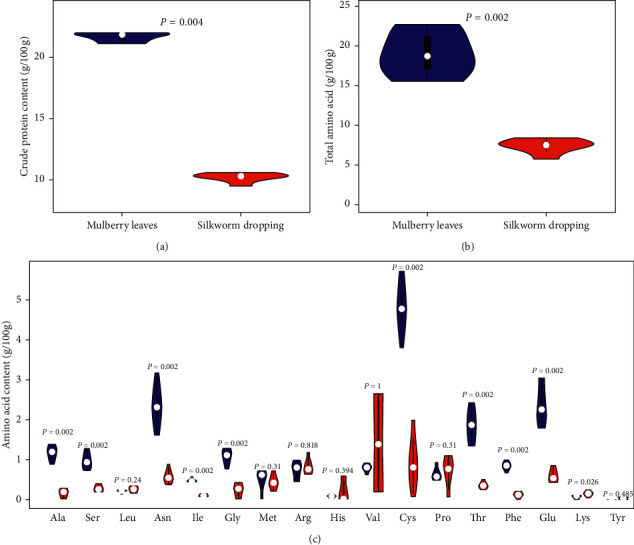
Crude protein and amino acid content in ML and SD. (a) Total protein content in ML and SD. (b) Amino acids content in ML and SD. (c) Violin plot of 17 amino acids proportions in ML and SD. The samples of ML were blue and samples of SD were red.

The protein has to be consumed as part of an otherwise nutritionally adequate diet to achieve the desired structure and function. Amino acids are required for body protein synthesis and nitrogen-containing compounds, such as hormones and neurotransmitters. The content of crude proteins and amino acids had a significant (*P* = 0.004 and *P* = 0.002) effect on ML and SD (Figures 2(a) and 2(b) and Table S2). The alanine, serine, asparagine, isoleucine, glycine, cysteine, threonine, phenylalanine, and glutamic acid contents of ML were higher than those of SD (*P* < 0.05) (Figure 2(c) and Table S2). However, lysine content was higher in SD (*P* < 0.05) (Figure 2(c) and Table S2).

### 3.3. Differentiated Components and Metabolic Pathway Analysis

**Figure 3:**
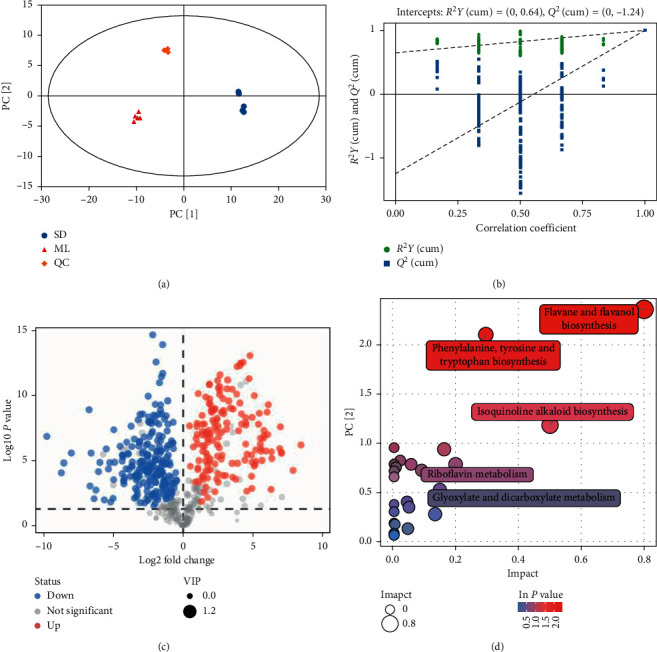
The metabolic analysis of ML and SD. (a) The score scatter plot of PCA model for ML, SD, and QC samples. (b) OPLS-DA model corresponding validation plots for ML and SD. (c) The differentiated compounds between ML and SD (VIP>1 and *P* value < 0.05). The dot size indicates the variable importance in the projection (VIP) value. Fold change: mean value of peak area obtained from the mulberry leaves/mean value of peak area obtained from the SD group. (d) Metabolic pathway analysis for differentiated compounds between ML and SD. Significantly changed pathways based on enrichment and topology analysis are shown. The *x*-axis represents the pathway impact, and the *y*-axis represents the pathway enrichment. Larger sizes and darker colors represent higher pathway impact values and higher pathway enrichment, respectively.

The metabolic profiles of samples from quality control (QC), ML, and SD were acquired by the validated UHPLC/Q-TOF MS methods in positive and negative ion modes (Figure S1). The results of each group were clustered using PCA (Figure 3(a)). All metabolite peaks were assigned by a self-established secondary mass spectrometry database. Approximately 604 components were identified in ML and SD (AnnoScore>0.9) (Table S3).

To clarify the metabolic changes of ML and SD, the metabolic differences were characterized by OPLS-DA. The samples from ML and SD (*R*^2^*X* (cum) = 0.816, *R*^2^*Y* (cum) = 1, and *Q* (cum) = 0.998) were unambiguously separated according to their difference in the entire metabolic profiles by OPLS-DA permutation test (Figures 3(b) and S2). These results indicated that the metabolic profiles of ML and SD were greatly perturbed after silkworm digestion.

To identify the differentiated metabolites contribution, the VIP was constructed by OPLS-DA and *P* value by Student's *t*-test. The 386 differentiated compounds were found between ML and SD (VIP>1 and *P* value < 0.05) (Figure 3(c) and Table S4). The content of 156 compounds in ML was higher than that in SD (Table S4). There were 83 mini-peptides among all of the differentiated compounds. The content of 66 mini-peptides was higher in ML than in SD, while the content of 17 mini-peptides was higher in SD than in ML. The KEGG pathways of differentiated compounds were analyzed. The flavonoid biosynthesis and phenylalanine as well as tyrosine and tryptophan biosynthesis pathways were the main differential compound synthesis pathways (Figure 3(d)).

### 3.4. Network Pharmacology Analysis

**Figure 4:**
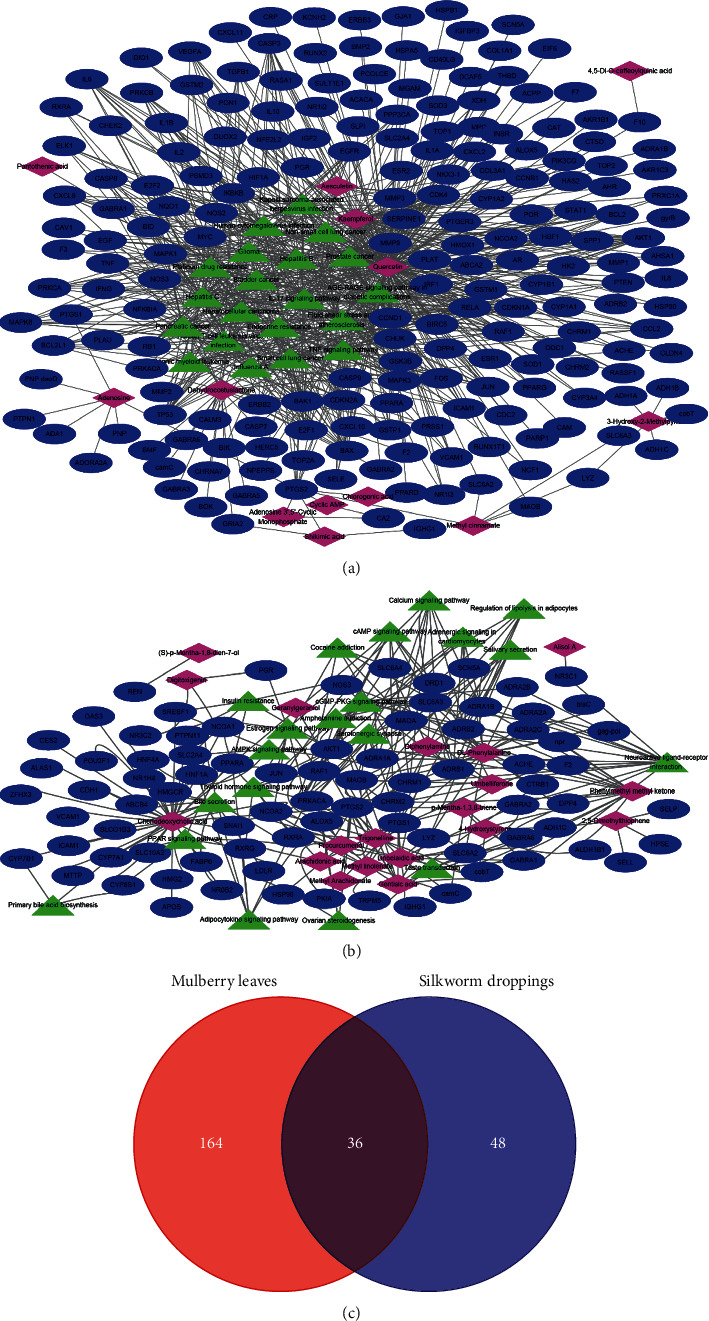
The components-target gene-pathway interaction network. (a) The components-target gene-pathway interaction network of higher content of components in ML. (b) The components-target gene-pathway interaction network of higher content of components in SD. (c) Venn diagram of target genes of differentiated compounds between ML and SD.

The 303 differentiated compounds except for 83 mini-peptides were searched in the TCMSP and SymMap database, generating information on 32 components (Figure 4). The content of 13 components was higher in ML than in SD, while the content of 19 compounds was higher in SD than in ML. The related target genes of 32 components in ML and SD were also searched. The compound-target-pathway network was constructed using Cytoscape software based on the degree of a topological parameter (Figure 4(a) and 4(b)).

**Figure 5:**
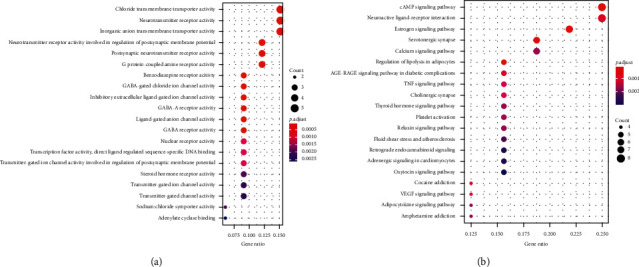
Go and KEGG enrichment of common target genes for the different compounds between ML and SD. (a) Significantly enriched GO terms; the emapplot of gene overlap enriched GO terms and gene correlation between most prominent GO terms in common target genes. (b) Significantly enriched KEGG pathways; the emapplot of gene overlap enriched KEGG pathways and gene correlation with most prominent KEGG pathways in common target genes.

**Figure 6:**
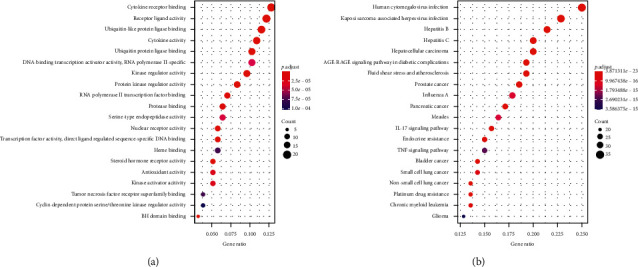
GO and KEGG enrichment of unique target genes of ML. (a) Significantly enriched GO terms; the emapplot of gene overlap enriched GO terms and gene correlation between most prominent GO terms in unique target genes of ML. (b) Significantly enriched KEGG pathways; the emapplot of gene overlap enriched KEGG pathways and gene correlation with most prominent KEGG pathways in unique target genes of ML.

**Figure 7:**
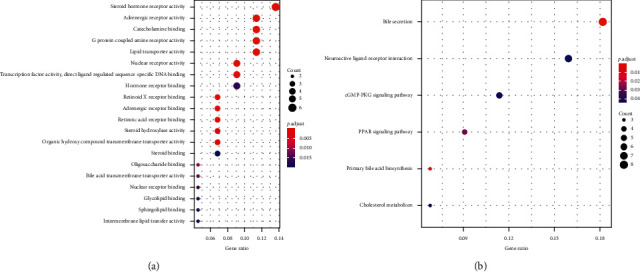
GO and KEGG enrichment of unique target genes in SD. (a) Significantly enriched GO terms; the emapplot of gene overlap enriched GO terms and gene correlation between most prominent GO terms in unique target genes of SD. (b) Significantly enriched KEGG pathways; the emapplot of gene overlap enriched KEGG pathways and gene correlation with most prominent KEGG pathways in unique target genes of SD.

The 36 target genes are common targets for the different compounds between ML and SD (Figure 4(c)). The enriched GO terms and pathways of common target genes are shown in Figure 5. The special target genes in ML were related to ubiquitin-protein ligase, cytokine, and kinase activity gene functions and involved in multiple viral infections and cancer pathways (Figure 6). However, the special target genes in SD were related to steroid hormone receptor activity, adrenergic receptor activity, and catecholamine binding gene functions and involved in neuroactive ligand-receptor interaction, bile secretion, and cGMP-PKG signaling pathway (Figure 7).

## 4. Discussion

ML and SD are commonly used in traditional Chinese medicine. The medical functions of both ML and SD are similar but different due to the digestion of silkworm. More than 50% of the constituents were different between MeOH extracted ML and SD by thin-layer chromatography (TLC) patterns [26]. The active ingredients in ML include organic acids, flavonoids, and alkaloids such as gallic acid, fumaric acid, chlorogenic acid, quercetin, and 1-Deoxynojirimycin (1-DNJ) [27–29]. 1-DNJ inhibits alpha-glycosidase involved in the hydrolysis of carbohydrates and prevents sugar from entering the bloodstream [30]. Also, the ML extract has been reported to inhibit cholesterol absorption in the intestine to have an antihyperlipidemic and atherosclerosis effect [31–33]. The polysaccharides of ML own antioxidant properties [34]. Mulberry leaf extract can resist hepatotoxicity induced by methotrexate [35]. SD can serve as a cheap source including chlorophylls, vitamins, and metal complexes of porphyrins [16]. SD extract (shengxuening tablet) has been used as an efficient oral iron supplement to IDA [36–38]. SD extract ameliorates various allergy symptoms by regulating Th1/Th2 immune response [39, 40].

Lipids are important and elemental nutrients for health and include cholesterol and fatty acids. Meanwhile, cavitating oil-water flows and oil viscosity affects the extraction efficiency of lipids in different containers [41]. In our research, the samples were extracted with petroleum ether in Soxhlet extractor. Lipid molecules are important components of membranes and mediators of multiple signaling pathways [42–44]. The saturated and unsaturated fatty acid can affect cardiovascular disease (CVD) progress by inflammatory and oxidative stress [45–47]. We found no significant difference in crude fat content between ML and SD, but there was a significant difference in the contents of some long-chain fatty acids (Figure 1 and Table S1). The C15 : 0 (pentadecanoic acid) fatty acids deficiency contributes to liver injury in nonalcoholic fatty liver disease (NAFLD) [48]. Oleic acid (C18 : 1) can affect embryo development by a metabolite of fatty acids [49]. The oleic acid owns anti-inflammatory activity as an alternative to treat inflammatory skin disorders [50]. The linolenic acids were potent antiglycation and advanced glycation end-products inhibition compounds [51]. Linolenic acid attenuates acetylcholine-induced relaxation by inhibiting nitric oxide-induced cGMP formation [52]. Besides, lipid might be regarded as an oral drug delivery system to provide solubility of the drug and avoid vessel embolization [53–55].

Proteins, made of amino acids, are responsible for nearly every task of cellular life to act as catalysts or tiny pumps and so forth. The protein nutrition impairs host immunity, especially the T-cell system [56]. The protein content of ML is significantly higher than those of other green leafy vegetables [57]. In our studies, we found that crude protein and amino acid content of ML was significantly higher than that of SD (*P* value < 0.05) (Figure 2). The content of lysine in SD was higher than that in ML (Figure 2(c)). This is due to the digestion and absorption in the silkworm digestive tract of proteins and amino acids. Lysine might induce humoral and cell-mediated inflammatory and immune responses to augmented healing of all types of wounds and induce angiogenic responses [58, 59]. The amino acid metabolism disorders induce an increase in the plasma amino acid concentration [60]. Although only 2% of the protein is made up of cysteine, cysteine is the major of posttranslational modifications [61]. Although cysteine is a nonessential amino acid, a lack of cysteine can cause oxidative stress to induce neurodegenerative diseases [62–65]. In our studies, cysteine has the highest content of amino acids in ML (Figure 2(c)).

The chemical metabolomics and network pharmacology can comprehensively characterize the Chinese materia medica and reflect multiple components and multiple targets [18, 66–71]. The comprehensive analysis of metabolomics and network pharmacology is consistent with the “holistic” perspective of TCM and the effect of Chinese materia medica. The differentiated components and metabolic pathways were analyzed to identify potential biomarkers to the unique medicinal properties of mulberry leaves and silkworm droppings. We found that the contents of kaempferol and quercetin involved in flavone and flavonol biosynthesis pathway in mulberry leaves were higher than those in silkworm droppings (Figure 3(d) and Table S4). The beneficial effects of flavonoids in ML are resisting cancer, as well as inflammatory and viral activities [72–74]. The content and compound species of benzene and substituted derivatives, carboxylic acids and derivatives, fatty acyls, and prenol lipids were more in silkworm droppings than in mulberry leaves (Table S4). These results suggest that the intestinal metabolism of silkworms increases the complexity of mulberry leaf compounds. However, the targets of many different compounds in silkworm sand are consistent with those in mulberry leaves (Figure 4(c)). Compared with silkworm droppings, the main target pathways of differential compounds in mulberry leaves are viral infection and cancer signaling pathways (Figure 6). These different biological pathways between mulberry leaves and silkworm droppings might be related to their different medical properties.

## 5. Conclusion

Pharmacodynamic substance bases and pharmacological targets of ML and SD were analyzed by metabolomics and network pharmacology. The fatty acids, amino acids, and flavonoids in SD were significantly changed compared with those in ML after digestion by silkworm intestines. The main pathway of mulberry leaf is enriched in antivirus and cancer, while the pathway of SD is enriched in hormone regulation and signal transduction pathways. These results might be related to the traditional Chinese medicinal properties of ML and SD and suggested that intestinal digestion and absorption of silkworm played an important role in the change of the pharmacodynamic substance basis and pharmacodynamic activity of ML and SD. This study would offer new insight into the biotransformation of Chinese materia medica.

## Data Availability

The data used to support the findings of this study are available from the corresponding author upon request.

## Abbreviations

TCM: Traditional Chinese medicine
1-DNJ: 1-Deoxynojirimycin
CVD: Cardiovascular disease
ESI: Electrospray ionization source
GO: Gene Ontology
IDA: Information-dependent basis
KEGG: Kyoto Encyclopedia of Genes and Genomes database
ML: Mulberry leaves
NAFLD: Nonalcoholic fatty liver disease
OPLS-DA: Orthogonal partial least squares discriminant analysis
PCA: Principal component analysis
PLS-DA: Partial least squares discrimination analysis
PUFA: Polyunsaturated fatty acid
QC: Quality control
SD: Silkworm droppings
TCMSP: Traditional Chinese Medicine Systems Pharmacology
TLC: Thin-layer chromatography
VIP: Variable importance in the projection.

## Supplementary Materials

Supplementary Materials Table S1: total fat and fatty acid content in mulberry leaves and silkworm droppings. Table S2: the crude protein and amino acids content in mulberry leaves and silkworm droppings. Table S3: the components were identified in mulberry leaves and silkworm droppings by UHPLC/Q-TOF MS. Table S4: the differentiated compounds were found between mulberry leaves and silkworm droppings (VIP>1 and *P* value < 0.05). Figure S1: the ion flow diagram of sample was extracted by NEG (A) and POS (B) mode. Figure S2: the score scatter plot of PCA, PLS-DA, and t-SNE model for mulberry leaves and silkworm droppings. (A) The score scatter plot of PCA model for mulberry leaves and silkworm droppings. (B) The score scatter plot of PLS-DA model for mulberry leaves and silkworm droppings. (C) The score scatter plot of t-SNE model for mulberry leaves and silkworm droppings.
