# Supplementary figures and images for: Differential Metabolomics and Network Pharmacology Analysis of Silkworm Biotransformation between Mulberry Leaves and Silkworm Droppings
Source: Evid Based Complement Alternat Med. 2021 Jun 29;2021:8819538. doi: 10.1155/2021/8819538 (PMC8263261; doi:10.1155/2021/8819538)

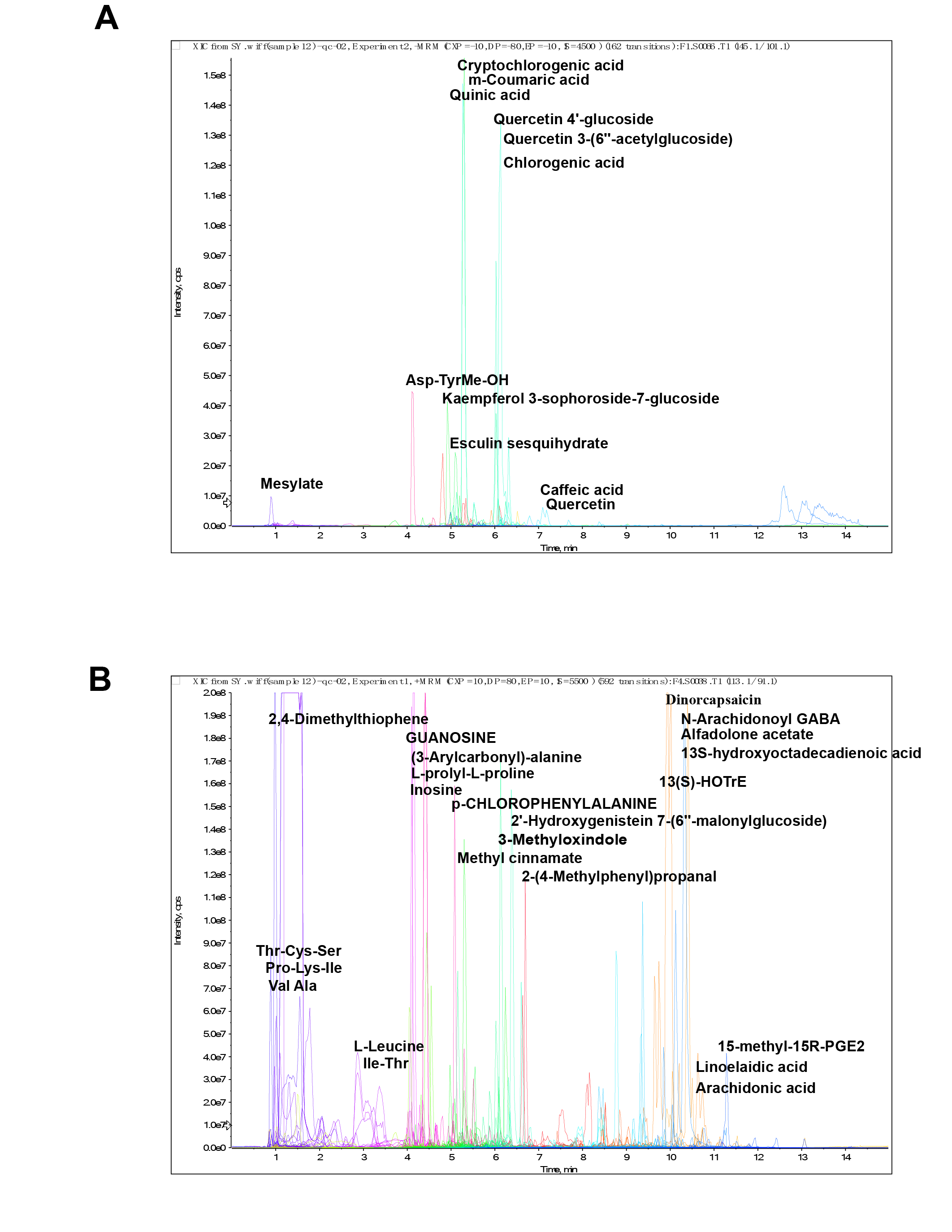

Supplement: Supplementary Materials — Table S1: total fat and fatty acid content in mulberry leaves and silkworm droppings. Table S2: the crude protein and amino acids content in mulberry leaves and silkworm droppings. Table S3: the components were identified in mulberry leaves and silkworm droppings by UHPLC/Q-TOF MS. Table S4: the differentiated compounds were found between mulberry leaves and silkworm droppings (VIP>1 and P value < 0.05). Figure S1: the ion flow diagram of sample was extracted by NEG (A) and POS (B) mode. Figure S2: the score scatter plot of PCA, PLS-DA, and t-SNE model for mulberry leaves and silkworm droppings. (A) The score scatter plot of PCA model for mulberry leaves and silkworm droppings. (B) The score scatter plot of PLS-DA model for mulberry leaves and silkworm droppings. (C) The score scatter plot of t-SNE model for mulberry leaves and silkworm droppings. [file 8819538.f1.zip › 8819538.f1/Figure 1S.png]

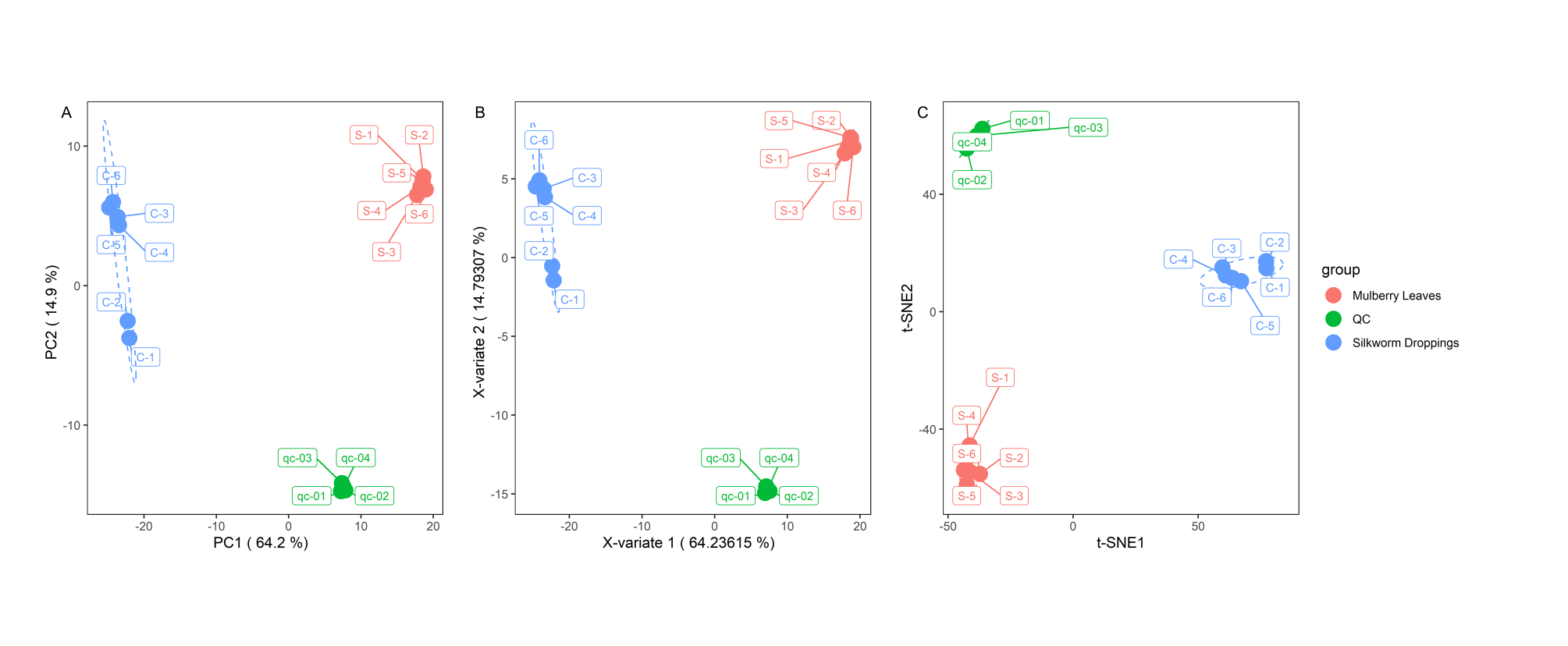

Supplement: Supplementary Materials — Table S1: total fat and fatty acid content in mulberry leaves and silkworm droppings. Table S2: the crude protein and amino acids content in mulberry leaves and silkworm droppings. Table S3: the components were identified in mulberry leaves and silkworm droppings by UHPLC/Q-TOF MS. Table S4: the differentiated compounds were found between mulberry leaves and silkworm droppings (VIP>1 and P value < 0.05). Figure S1: the ion flow diagram of sample was extracted by NEG (A) and POS (B) mode. Figure S2: the score scatter plot of PCA, PLS-DA, and t-SNE model for mulberry leaves and silkworm droppings. (A) The score scatter plot of PCA model for mulberry leaves and silkworm droppings. (B) The score scatter plot of PLS-DA model for mulberry leaves and silkworm droppings. (C) The score scatter plot of t-SNE model for mulberry leaves and silkworm droppings. [file 8819538.f1.zip › 8819538.f1/Figure 2S.png]
